# Instruments to assess integrated care: a systematic review

**Published:** 2014-09-25

**Authors:** Anne Marie Lyngsø, Nina Skavlan Godtfredsen, Dorte Høst, Anne Frølich

**Affiliations:** Department of Integrated Healthcare, Bispebjerg University Hospital, Bispebjerg Bakke 23, DK-2400 Copenhagen, Denmark; Department of Respiratory Medicine, Hvidovre University Hospital, Kettegaard Allé 30, DK-2650 Hvidovre, Denmark; Department of Integrated Healthcare, Bispebjerg University Hospital, Bispebjerg Bakke 23, DK-2400 Copenhagen, Denmark; Department of Integrated Healthcare, Bispebjerg University Hospital, Bispebjerg Bakke 23, DK-2400 Copenhagen, Denmark

**Keywords:** integrated care, systematic literature review, measurement instruments, organisational elements

## Abstract

**Introduction:**

Although several measurement instruments have been developed to measure the level of integrated health care delivery, no standardised, validated instrument exists covering all aspects of integrated care. The purpose of this review is to identify the instruments concerning how to measure the level of integration across health-care sectors and to assess and evaluate the organisational elements within the instruments identified.

**Methods:**

An extensive, systematic literature review in PubMed, CINAHL, PsycINFO, Cochrane Library, Web of Science for the years 1980–2011. Selected abstracts were independently reviewed by two investigators.

**Results:**

We identified 23 measurement instruments and, within these, eight organisational elements were found. No measurement instrument covered all organisational elements, but almost all studies include well-defined structural and process aspects and six include cultural aspects; 14 explicitly stated using a theoretical framework.

**Conclusion and discussion:**

This review did not identify any measurement instrument covering all aspects of integrated care. Further, a lack of uniform use of the eight organisational elements across the studies was prevalent. It is uncertain whether development of a single ‘all-inclusive’ model for assessing integrated care is desirable. We emphasise the continuing need for validated instruments embedded in theoretical contexts.

## Introduction

Integrated care has been on the health-care agenda since the 1970s. During the past two decades, there has been a rapidly growing interest in how to develop better and more cost-effective health systems focusing on its impact [1]. This increasing focus on integrated care as a means of improving the performance of health systems is widespread in Europe, North America and other parts of the world [2]. In essence, integrated care can be seen as a demand-driven response to what generally ails modern-day health care [3, 4].

The proportion of elderly persons above 65 years of age is increasing in Western countries, and the demographic trend seems set to continue. This is coupled with an increase in the proportion of individuals with one or more chronic conditions, and, accordingly, the delivery of appropriate care for these persons requires a paradigm shift from episodic, short-term interventions, characteristic of acute conditions, to long-term, comprehensive care. Those with chronic conditions often require complex and continuous interventions spanning professions, sectors and political levels [3, 5, 6].

The need for integrated care contrasts strongly with the accelerating specialisation and division of labour within the health-care system predominantly arising from medical development. This development necessitates health-care personnel acquiring more in-depth medical knowledge, usually at the cost of knowledge of closely related specialities. Despite specialisation having several advantages, including better and safer job performance, it also has disadvantages. First, organisational fragmentation complicates the management of organisational units. Second, professionalisation combined with decentralisation reinforces a cultural fragmentation that preserves ‘tribal values’, making it difficult to develop and share common values among the health-care personnel. Consequently, optimal collaboration and coordination between professionals and sectors in delivering integrated care have become key in providing high-quality care. To guide the further implementation of integrated care models, there is an urgent need for evaluations that can help assess whether the proposed models support integration and high-quality care. While such evaluations are important for practitioners and researchers, they are crucial for the managers charged with the process of implementing and sustaining integrated care [7, 8].

Different measurement instruments have been developed to measure the level of integration of diverse forms of services and networks within the health-care system [8, 9]. To support development of evidence in the area of integrated care, systematic literature reviews are central for various reasons. First, knowledge of existing instruments can avoid new ones being developed, particularly if a measurement instrument exists that can be transferred and adapted to new settings. Second, if new instruments must be developed, it is essential to obtain input about what these should or should not contain and how they should appear. Finally, there is mounting evidence of the potential of systematic reviews to serve as tools for evidence-based decision making for health planners and policy-makers [10].

A systematic literature review of health science – and business databases up until January 2007 revealed a substantial lack of high-quality studies and standardised instruments to evaluate integration outcomes [11]. Further, despite numerous papers on the subject, no universal definition or concept of integration was found. Another recent review performed a literature search up until April 2008 revealing 24 different measurement instruments of integrated care in 24 published articles [12]. The authors suggest various measurement criteria to guide future research and highlight the importance of validating and simplifying the existing instruments. Moreover, it is central that a measurement instrument cover the most important organisational elements supporting integrated care in chronic conditions. However, to our knowledge, no previous review has analysed the influence of the organisational elements in the conceptualisation of integrated care. Accordingly, to support the review process, we searched for evidence on important organisational elements.

## Purpose

The purpose of this systematic review is to identify studies on currently available instruments to measure the level of integrated care across health-care sectors.

The following research questions were set up for the review:

1. Which measurement instruments exist for measuring the level of integrated care across health-care sectors?
2. What organisational elements are most commonly included in the published measurement instruments?
3. How do the identified organisational elements correlate with those stated as important for creating integrated care elsewhere in the literature?
4. What are the similarities and differences of the published measurement instruments?

## Conceptual framework

Despite the interest in integrated care, conceptual diversity within the field is vast and is a barrier to understanding and creating integrated care, both in theory and in practice, and to monitoring the processes of integration [13]. In this review, the definition of integrated care stated by Kodner and Spreeuwenberg [1] is used*:* ‘Integration is a coherent set of methods and models on the funding, administrative, organisational, service delivery and clinical levels designed to create connectivity, alignment and collaboration within and between the cure and care sectors. The goal of these methods and models is to enhance quality of care and quality of life, consumer satisfaction and system efficiency for patients with complex, long-term problems cutting across multiple services, providers and settings. The result of such multi-pronged efforts to promote integration for the benefit of these special patients groups is integrated care’ [1]. Even today, the knowledge of the factors affecting integrated care is incomplete, making it difficult to state what actually creates integrated care and, hence, how best to monitor the processes of care and with which indicators [13]. As highlighted in the definition above, this review builds on the idea that integrated care is not only created by the presence of a single mechanism but by the combination of numerous integrated activities operating at different levels. This belief is consistent with an early consensus in the integration literature, stating that a comprehensive measurement approach needs to consider multiple dimensions, components and perspectives on integrated care [9].

As highlighted in the aforementioned definition of integrated care, integration is a means to improve the quality of health services (in relation to quality of care and quality of life). With this in mind, Donabedian's framework on quality of medical care was used in the further selection process of measurement instruments. The framework is divided into the following aspects: structure, process and outcome; with the first two dimensions considered as main features of integration [3, 14]. The outcome of medical care, expressed in terms of hospital readmission rates, functional status level and survival rates, has been frequently used as an indicator of quality of medical care. In the literature on integrated care, the purpose is often to test whether case management and disease management programmes can lower costs and improve patient outcomes. The aims of these studies are not to integrate the full range of health-care services and measure to what extent this has been done, but to investigate whether these programmes can lower costs and improve patient outcome [15]. For this reason, only instruments measuring structural and process aspects were included in the review. Structure is concerned with such things as the adequacy of facilities and equipment: the qualifications of medical staff and their organisation [14]; process concerns how the work is done: work routines, communication between staff members and user involvement. Care coordination is a way of achieving integration at the micro-level by ensuring that service users experience seamless care. Despite the importance of the perspective, it gives limited insight into the integration of services at both the system and organisational level. In addition to the structure and process, the review included instruments with a cultural perspective that also take into account the meaning of shared beliefs, norms and values [16].

## Methods

### Inclusion criteria

As underlined above, integrated care is a nested concept inasmuch as it can be defined and analysed from many perspectives. In addition, the strategies used to create integrated care depend on the characteristics of the patient group and the specific challenges patients face in obtaining appropriate, quality care. In this review, the focus was on measurement instruments directed towards individuals with an ongoing treatment need.

Thus for review inclusion, the respondent group had to be persons working within the health-care system, either holding an administrative position or being part of the front-line staff.

Articles analysing only patients’ perceptions of coordination were not included.

For review inclusion, each article had to meet the following inclusion criteria:

- Include a measurement instrument measuring the level of integration across health-care sectors (articles focusing on only collaboration within health – and social-care sectors, such as primary care, hospitals or community-based services, were excluded).
- Include a measurement instrument focusing on the combination of numerous integrating activities (articles focusing on only the presence and use of clinical guidelines were excluded).
- Include an instrument measuring structural, process and/or cultural aspects of integrated care.
- Include a measurement instrument focusing on the organisation of the treatment of individuals with an ongoing treatment need.
- Include a measurement instrument with a respondent group consisting of persons working within the health-care system either holding an administrative position or being part of the front-line staff.

### Search strategy

The health science literature (PubMed, CINAHL, PsycINFO, Cochrane Library and Web of Science) for the years 1980–2011 was searched for relevant articles. In addition, personal emails were sent to experts in the field in search of additional articles or reports. Publications written in English, Danish, Swedish and Norwegian were included. Articles written in other languages would have been included if an English abstract existed. To identify relevant search terms, systematic reviews and other articles on integrated care were searched [1–3, 8, 9, 11]. The use and combination of words and Medical Subject Headings terms for PubMed are shown below. A similar search strategy was used for all databases. All search strategies and databases were developed and searched together with a medical research librarian.

### The review process

**Figure 1.**
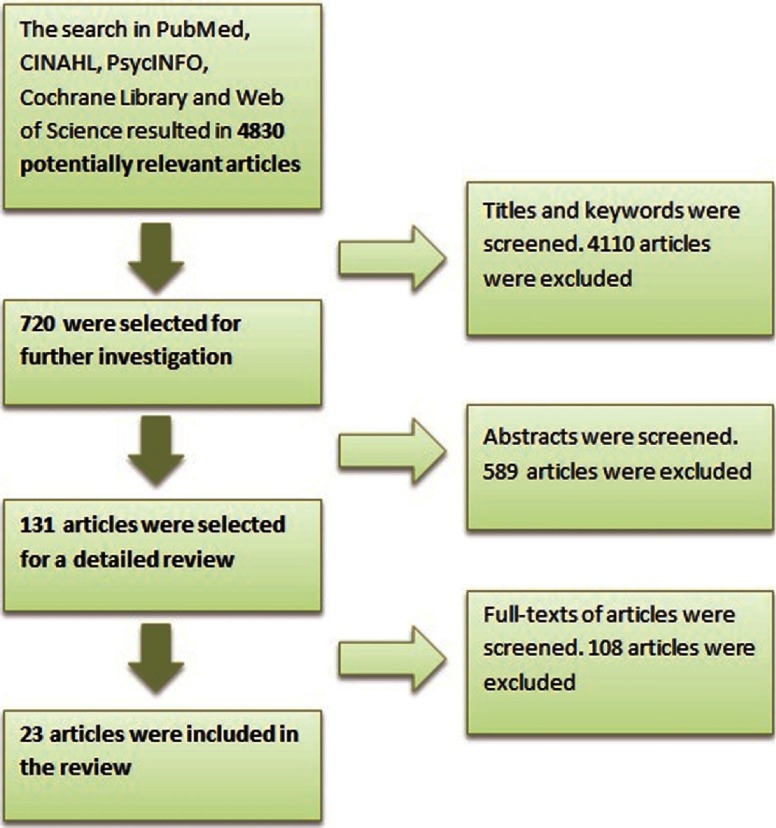
The review process.

From the five databases, 5123 articles were identified; with 4830 when duplicates were removed. After reviewing the titles, 720 abstracts were reviewed by the investigator and a coinvestigator; from those abstracts, 131 articles were selected for full review (Figure 1).

## Results

### Shared features and differences

The inclusion criteria were met by 23 articles. To systematise our findings, we extracted details from each article using a set of criteria outlined from the literature [17, 18]. We considered a criterion fulfilled if it was explicitly stated in the article.

Table 1 covers reference [19–41] and includes name of first author and year of publication, research objective, construct of interest, type of measurement instrument, patient group and respondent group.

In general, we found no unified or commonly agreed-upon measurement instrument (Table 1). Instead, the diversity of approaches to measure integration across health-care sectors was wide for most of the analysed criteria. When looking at the criterion *construct of interest*, it becomes clear how complex it is and how many different aspects and levels it includes. Thus, functional, clinical and system integration are all being measured in the instruments identified.

The methods used to measure integration varied widely between questionnaire survey data, inpatient data/clinical files analysis and different qualitative methods such as interviews, observations and workshops. However, the combination of methods is that most widely used.

For the patient group, there is a difference in the identified instruments regarding level of specification. Some instruments have only patients with a specific disease as target population, whereas others incorporate a number of hospitals or organised delivery systems. This is important to bear in mind when discussing the possibilities of transferring and adapting the instruments to new settings.

In line with the inclusion criteria, the respondent groups also vary and include policy-makers, system and operating unit managers, administrators, physicians/front-line staff and representatives or coordinators from each service within the different sectors.

Table 2 shows a further analysis of the identified measurement and includes defined construct; theoretical framework; defined level of analysis; and structural, process and cultural aspects. These criteria are derived from Strandberg-Larsen et al. 2009 [12].

The multi-dimensionality in the construct of interest from Table 1 underlines the need for a clear definition of the construct being measured within each article and the presence of a theoretical framework. As shown in Table 2, almost all the measurement instruments have defined the construct being measured, but only 15 articles have explicitly described the use of a theoretical framework. The level of analysis is stated in all articles. When looking at the different aspects measured, most studies include both structural and process aspects, whereas the inclusion of cultural aspects was present in only 7 of the 23 measurement instruments. Overall, only four instruments described all six criteria defined as central for a measurement instrument [23, 26].

### Organisational elements

In relation to the second aim of this review, we identified eight different organisational elements within the 23 measurement instruments. Table 3 shows each of the organisational elements in the left column and presents a further description of their content in the right column. When compared with other reviews in the field of integrated care, the elements are very similar.

To further analyse the use of each element, all articles were screened. Table 4 shows which elements are captured in each measurement instrument identified and also provides an overview of which elements are measured the most often. Most instruments contain items covering three or fewer of the organisational elements; however, a few capture six or more. The three elements used the most are IT/information transfer, commitment and incentives, and clinical care, covering such things as teams of multidisciplinary professionals, case management and clinical guidelines.

## Discussion

This review identified 23 measurement instruments that aimed to fulfil the crucial role of measuring the impact of integrated care models focusing on the level of integration based on central organisational elements. Apart from identifying the measurement instruments available, the purpose of the review was to elucidate the organisational elements that most commonly appear and that are measured within the published instruments. As in other evidence-based literature overviews in this expanding research field, it was not possible to identify a uniform instrument measuring integrated health care across different delivery systems.

### The identified measurement instruments

The number of instruments identified in this review may seem low considering the growing, widespread enthusiasm for integration and focus on measuring health system performance. The review by Strandberg-Larsen et al. found 24 instruments using a slightly different search strategy, which included grey-zone literature such as academic working papers and ministerial reports [12]. About half the instruments presented in our review are also included in the aforementioned paper. However, due to the inclusion criteria and our focus on the organisational elements, we retrieved some instruments not previously discussed in reviews. Although the development of new instruments has intensified since 2000, it is still limited, and information related to implementing and evaluating integration-centred initiatives remains a relatively new area in need of further investigation on how best to capture the process of creating integrated care. Many of the identified instruments build on the same theoretical framework, and the question is whether future research will continue to build on these frameworks or develop new ways of approaching the field.

### Important elements in creating integrated care

As shown in Table 1, a diverse array of concepts characterises the field and highly affects the content of the 23 instruments identified. Some organisational elements are more frequently measured across the different instruments than others, but to state which of these is the most important in the process of creating integrated care is still a complicated task. Each element proposed represents a hypothesis that has to be tested empirically. Some of these have already been proven to explain a positive variation in the process of building integrated care, but there remains a lack of evidence regarding which ones weigh higher than others.

The organisational elements of importance to integrated care identified in this review are comparable to those in other reviews in the field. Suter et al. 2009 conducted a review with the aim of summarising the current research literature on health-system integration. It highlighted 10 principles that were frequently and consistently presented as key elements for successful integration in the reviewed literature [10]. Thus, just as Suter and colleagues, we find that the following elements are important for building health-system integration independent of the health-care context or patient population served: IT/information transfer, organisational culture and leadership, commitments and incentives to deliver integrated care, clinical care (teams, case management, clinical guidelines and protocols), financial incentives, quality improvement/performance measurement and patient focus. Our findings are also in line with Kodner and Spreeuwenberg's discussion paper on integrated care [1]. In their paper, they state that a continuum of strategies – from the macro to the micro – is available to foster integrated care and address problem areas in five important domains: funding, administrative, organisational, service delivery and clinical [16]. The list of methods and instruments listed under each domain in their article is in keeping with the elements listed in Table 3 in this article. However, this is not surprising since Kodner and Spreeuwenberg's definition of integrated care was used as a conceptual framework for our study. Considering the conceptual framework used in this review, it is clear that this in itself articulates areas of importance when identifying indicators of performance in relation to integrated care. Though, the definition of integrated care used in this review states that *‘*integration is a coherent set of methods and models on the funding, administrative, organisational, service delivery and clinical levels’ and Donabidian's framework focuses on the importance of ‘structural and process aspects’. Nevertheless, there is a great need for further research in this area. Even though the conceptual understanding of integrated care has developed, the concept remains broad, making it difficult to outline which indicators of performance and/or measures of quality are the most important and valid. As a conclusion, this means that the field of integrated care still holds challenges in supporting implementation and quality improvement because the outcome measures remain difficult to define.

In this review, the three key factors to the implementation of integrated care are IT/information transfer, commitments and incentives to deliver integrated care and clinical care (teams, clinical guidelines and protocols) (Table 4). These three factors were those represented the most strongly in the measurement instruments. In terms of adoption, it can be discussed whether the strong representation means that these factors are more important than the rest of the identified organisational elements in the process of creating integrated care. However, no studies have shown some factors to have a stronger impact than others. The reason that the three factors are included more often than others may be an expression of the fact that these factors are, in general, well implemented. Moreover, these factors may have been implemented more often in the instruments as they may be easier to measure than other elements, such as organisational culture.

In relation to how to measure the eight factors, this is done differently in the identified instruments. In relation to this, the concept of integrated care and its many definitions still play a major role. As long as there is disagreement about what the concept covers, it will be difficult to reach consensus about how to measure each factor and, hence, to assess the quality of an instrument trying to outline the level of integration based on that specific factor. Further, there is no evidence on how the different factors influence each other when they are present in combination with each other.

### The importance of the different elements and the opportunity of cross-national replication

Common to the measurement instruments identified is the purpose of measuring more than one element, and in this respect, it is highly relevant to know the importance of each element to actually calculate sum scores that make sense and are useful for further operation. Additionally, the importance of the different elements may well vary depending on the country in which the evaluation is done. A related question is then whether these different instruments can effectively be replicated in the health-care systems of countries other than those where they are developed. Even though the Western countries are facing similar challenges regarding the growing number of persons with one or more chronic conditions, there are significant differences in the way chronic care services are organised, paid for and delivered. Accordingly, even though an instrument in one country has shown to be useful in tracking system progress, setting concrete goals and assessing progress towards them, a new validation is necessary for each instrument applied in a new setting [18].

### The inclusiveness of the instruments

It is debatable whether a measurement instrument consisting of several elements is necessarily better than one covering only a limited number of elements. Integrated care is a complex concept with numerous embedded meanings, and building integrated care requires many different procedures at different levels of the system. Taking this into consideration, it seems the most appropriate to use instruments covering virtually all these procedures; nevertheless, being precise and explicit about the purpose and limitations of a certain evaluation is, perhaps, more important. Without this, it becomes problematic to judge whether the choice of measurement instrument actually works within the given context.

### Strengths and limitations of the review

The strengths of this systematic literature review include the broad search of all concepts related to the specific construct of integrated care, the identification of organisational elements important for the establishment and evaluation of integrated care and the systematic identification of the organisational elements present in each published instrument.

That the review focused on only literature published in the scientific health-care literature can be a limitation of the study. As Armitage et al. 2009 state, the inclusion of business literature could have proved fruitful [11]. Nevertheless, we found the inclusion criterion of ‘instruments focusing on the organisation of the treatment of people with an ongoing need of treatment’ crucial in the selection of databases as these patient groups often need more advanced integration of services; hence, instruments from the business literature did not seem suitable for this review.

Another limitation to the study is that the review considered integration only within the health-care sector. Thus, it did not focus on the institutional division between health and social care and the central need for integration between these sectors. Moreover, it did not take into account the role of third sector organisations. Many persons with complex needs, long-term conditions and terminal illness need to access different health-care, social care and even housing and other services. The evidence clearly shows that these services can be fragmented, and those who need to rely on them often find that they are difficult to access and that there are inadequate links between them. To focus on only integration within the health-care sector may therefore seem inadequate. However, intra-organisational integration within each sector is in itself a marked achievement and is highly important in the process of creating integrated care between health, social care, public health, other local services and the third sector. A review focusing on only measurement tools to measure integration within the health-care system should, therefore, not necessarily be seen as inadequate.

## Conclusion

This systematic literature review identified 23 measurement instruments developed to measure the level of integration across health-care sectors. As also stated by others, it has not been possible to identify a uniform instrument. Instead, the diversity of approaches to measure integration across health-care sectors seems wide and the organisational elements measured in each of the instruments vary. In this review, eight organisational elements were identified within the published instruments, each element consisting of a number of sub-components.

The diversity between the instruments identified is first a consequence of the lack of a clear and common understanding of the concept of integrated care. Second, the diversity arises from organisational variations within the health-care systems the instruments have been developed to measure. Both courses are central for decision-makers and researchers to take into account when planning and carrying out evaluations of integration within any given health-care system.

This review provides a list and detailed evaluation of some of the current measurement instruments within the field of integrated care. As other reviews within this area, this review is useful as a core of current evidence for further exploration and development, both theoretical and methodological. The explicit assessment and evaluation of the organisational elements is also important for decision-makers and planners as an indication of which strategies and processes to prioritise and establish. Evidence remains lacking on how the various organisational elements should be weighed against each other, and variations across countries hamper the assessment. The purpose of assigning weighting factors is to aid the process of establishing work priorities, and in the process of evaluating the level of integration within a given health-care system, they should be part of the calculation used to determine an accurate overall performance rating.

The complexity of health-care systems and delivery of services makes integration a difficult task. To guide further research in the process of achieving higher integrated health-care systems, we recommend research focusing on the following:

- Further elaboration on the concept of integrated care
- Case studies that involve closer assessment of the importance of the organisational elements in the process of creating integrated care systems
- Thorough and transparent research on how the various organisational elements must be weighed against each other
- Discussion papers on the challenge of replicating measurement instruments across different health-care settings
- Guidelines on how best to develop measurement instruments that can more effectively be replicated in the health systems of other countries
- Further validation and development of the already existing measurement instruments.

That the development of new instruments has intensified since 2000 but is still limited, shows that information related to implementing and evaluating integration-centred initiatives remains a relatively new area needing further investigation on how best to capture the process of creating integrated care.

## Figures and Tables

**Table 1.**
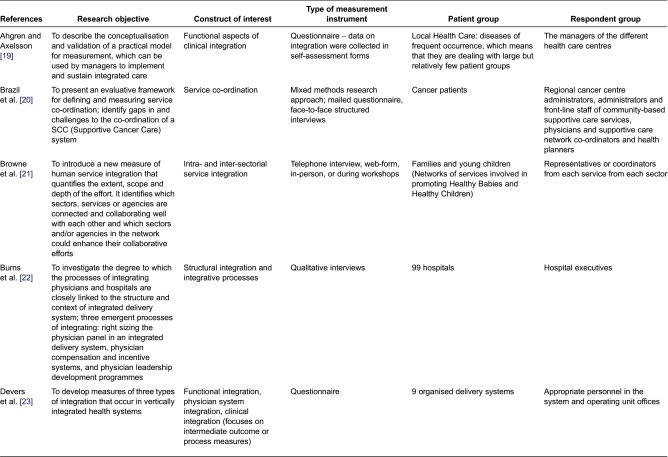

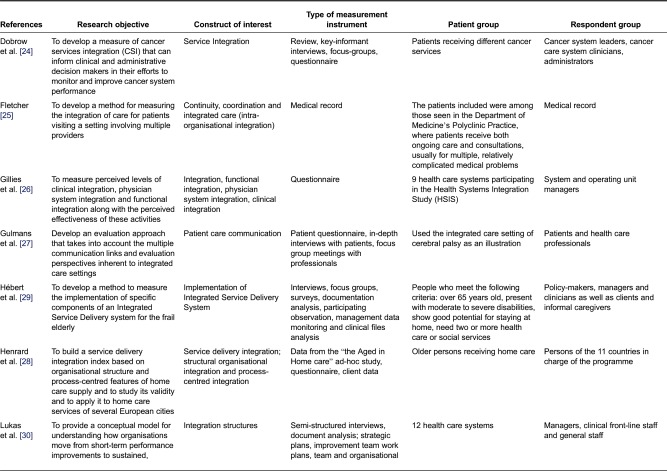

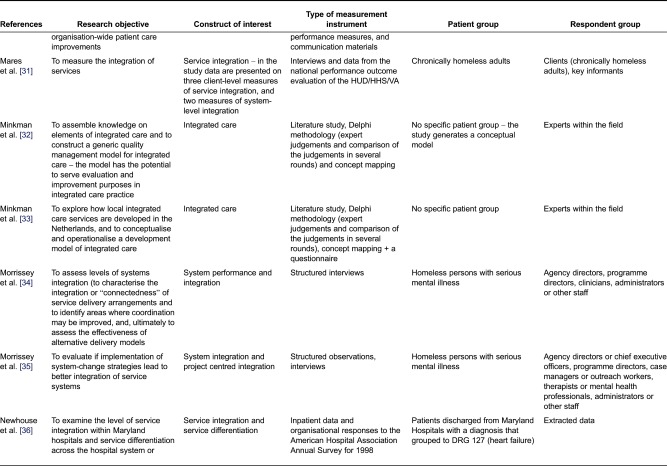

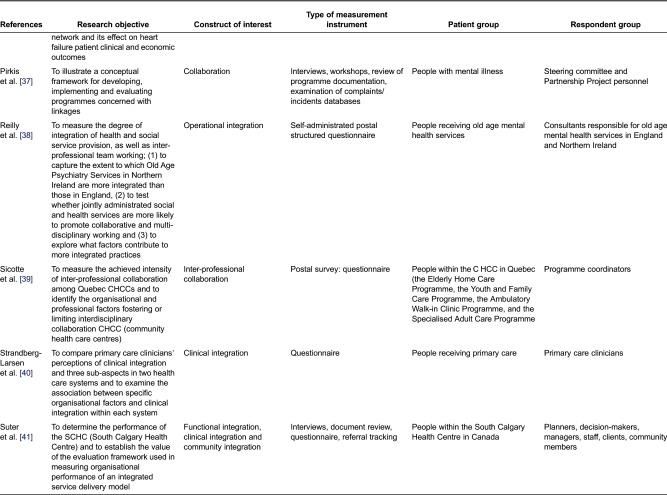
Measurement instruments published in scientific journals for measurement of integrated health care or related concepts

**Table 2.**
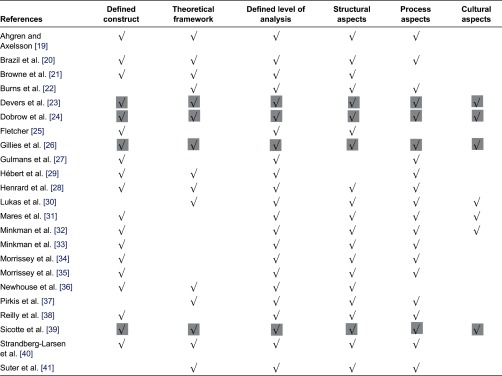
Overview of the criteria met for each of the identified measurement instruments

The four instruments meeting all criteria are highlighted in the table.

**Table 3.**
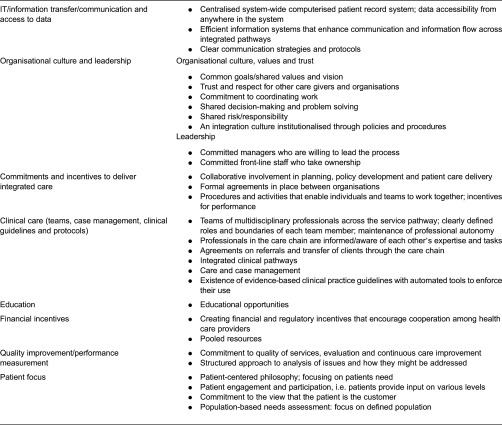
Essential organisational elements in building integrated care

**Table 4.**
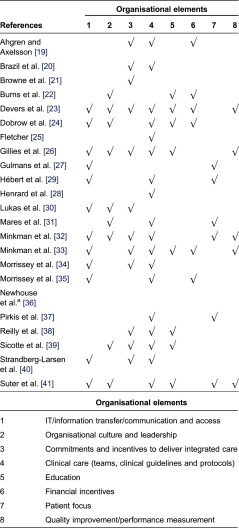
Overview of which organisational elements are measured in each of the identified measurement instruments

^a^Integration is expressed as “the percent of services available at the hospital level, calculated by dividing the number of services offered by the total available services”. No organisational elements are explicitly stated in the study.
